# Comparison Between the Effects of Ringer`s Lactate and Hydroxyethyl Starch on Hemodynamic Parameters After Spinal Anesthesia: A Randomized Clinical Trial

**DOI:** 10.5812/aapm.7850

**Published:** 2013-01-01

**Authors:** Mehdi Fathi, Farnad Imani, Marjan Joudi, Vahid Goodarzi

**Affiliations:** 1 Department of Anesthesiology, Emam-reza Hospital, Mashhad University of Medical Sciences, Mashhad, Iran; 2 Department of Anesthesiology and Pain Medicine, Rasoul Akram Medical Center, Iran University of Medical Sciences (IUMS), Tehran, Iran; 3 Depatment of Surgery, Mashhad University of Medical Sciences, Mashhad, Iran

**Keywords:** Hetastarch, Ringer`s Lactate, Anesthesia, Spinal

## Abstract

**Background:**

Hypotension during spinal anesthesia is common and can lead to severe injuries and even death. Administration of crystalloid fluids is advised to prevent occurrence of hypotension; however its effectiveness is still the matter of arguments.

**Objectives:**

This study was designed to compare the effects of Ringer`s lactate and hydroxyethyl starch 6% on hemodynamic parameters after spinal anesthesia in patients undergoing orthopedic surgeries on lower limbs.

**Patients and Methods:**

This randomized clinical trial was performed in Rasoul Akram Hospital, Tehran, Iran. 60 patients undergoing elective femoral fracture surgeries with spinal anesthesia were included in this study. Fitted patients were randomly divided into two equal groups. After entrance to the operation room and before spinal anesthesia, patients' hemodynamic parameters including systolic blood pressure (SBP), cardiac output (CO), and cardiac index (CI) were evaluated using monitoring electro-velocimetry set. In both groups, spinal anesthesia was performed using needle no. 25 and 3 mL of marcaine 0.5% in the sterile situation. None of the treatment group was aware of investigated group during the study.

**Results:**

The baseline values of mentioned variables did not show a significant difference between two groups using t-test (*P* > 0.05). Also SBP, CI, and CO after intervention was not significantly different between two groups using t-test (*P* > 0.05).

**Conclusions:**

The result of present study on patients undergoing femoral fracture surgeries who received Hetastarch or Ringer`s lactate solutions showed that Hetastarch was not significantly more effective in compensation of hypotension induced by spinal anesthesia.

## 1. Background

Hypotension during spinal anesthesia is common and can lead to severe injuries and even death (1-3) Administration of crystalloid fluids is advised to prevent occurrence of hypotension; however its effectiveness is still the matter of arguments (4, 5). Crystalloid solutions have short half-life and weak impacts on increasing plasma volume; this can explain the incidence of hypotension during spinal anesthesia in spite of administration of crystalloid fluids. Excess administration of crystalloid fluids also can lead to decline in oxygenation which increases the risk of pulmonary and peripheral edemas. Colloids are the first choice of pre-holding treatment in Europe (6). Colloid solutions remain more in circulation and seem to be an appropriate replacement for crystalloid solutions. Results of previous works showed that administration of colloid solutions before spinal anesthesia had a considerable effect on decreasing incidence of hypotension (7). Some references emphasize that preloading of patients, regardless the type of liquid, should be in some extent to improve cardiac output (CO) effectively and prevent from hypotension (8). These days, there are growing tendencies to use Hetastarch. This may be due to the fact that once crystalloids are used, two to four times volume is needed (8, 9). Several studies about electro-velocimetry set monitoring revealed reliability of its outcome compared to invasive methods for cardiac status assessment like transpulmonary thermodilution (10-12) and transesophageal electrocardiography (13). Moreover, this assessment method had a good correlation with golden standard method for cardiac index (direct oxygen FIK method) (14). Function of this equipment which is based on electrical bioimpedance and called "impedance cardiography" (ICG), "electrical cardiometry velocimetry" (ECV), or "bioreactance" (BR), measures a time-dependent electrical waveform modulated by blood flow through patient's thorax. Hetastarch is a colloid solution that is used commonly for increasing plasma volume in traumatic shock and sepsis. Nevertheless, effects of patient`s preload with colloid solutions on prevention from hypotension due to spinal anesthesia and other cardiac indexes like cardiac output (CO), heart rate (HR), cardiac index (CI), systemic vascular resistance (SVR), stroke volume (SV), and index of cardiac contractility (Icon) have not been assessed in patients undergoing orthopedic surgeries.

## 2. Objectives

This study was designed to compare the effects of Ringer`s lactate and hydroxyethyl starch 6% solutions on hemodynamic parameters after spinal anesthesia in patients undergoing femoral fracture surgeries.

## 3. Patients and Methods

This randomized clinical trial was conducted on 60 patients who underwent elective femoral fracture surgeries with spinal anesthesia in Rasool Akram Hospital, Tehran, Iran. Inclusion criteria were patients undergoing elective femoral fracture surgeries with spinal anesthesia, age 20-60 years, and physical status I and II based on the American Society of Anesthesiologist (ASA). Exclusion criteria were age < 20 or > 60 years, any relative or absolute contraindication of spinal anesthesia, and other conditions that could have an effect on hemodynamic responses like diabetes, essential hypertension, opium addiction, and heart disease. Fitted patients were randomly divided into two equal groups (each consisted of 30 patients). For randomization, we prepared numbers in one packet. This study was double blinded and none of patients, residents, or anesthesiologists knew about the groups. After the entrance to operation room and before intravenous fluid preloading, patients were evaluated using monitoring electro-velocimetry set (electrical velocimetry, Aesculoc, Osypka Medical, Germany). Standard monitoring devices such as electrocardiography, pulse oxymetry, and non-invasive blood pressure monitoring were used for all patients. Patients in group I received 10 mL/kg/IV from Ringer`s lactate solution 30 minutes before spinal anesthesia (Ringer`s lactate group), and 10 mL/kg/IV from hydroxyethyl starch 6% solution was administrated for patients in group II (Hetastarch group) 30 minutes before spinal anesthesia. In both groups, spinal anesthesia was performed through intervertebral space L2-L3 or L3-L4 using needle no. 25 and 3 mL of hyperbaric bupivacaine 0.5% in the sterile situation. It was performed in lateral decubitus position; patient's position during operation was supine and the bed was laid on the neutral position. The surface anesthesia was determined by existence of different parts of thermal sensations via touching the skin by moist cotton. This showed that the anesthesia did not exceed over sixth vertebrae of thoracic, and none of patients needed vasopressor. The range of height of patients was between 160cm and 180cm. In this study, hypotension was considered as systolic blood pressure below 80mm Hg below which the treatment was prescribed. Duration of surgeries was below three hours. Before beginning of surgery and in each 10 min intervals up to its end, hemodynamic variables including heart rate (beat/min), systolic and diastolic blood pressures (mm Hg), stroke volume (Lit), cardiac output (Lit/min), cardiac index (Lit/m2), index of contractility (mm Hg/s), and systemic vascular resistance (Dynes-sec/cm-5) were measured and noted in specific questionnaire for each patient. None of the treatment group was aware of investigated group during the study.

### 3.1. Statistical Analysis

Results were reported as mean ± standard deviation (SD) for quantitative variables, and percentages for categorical variables. The values of hemodynamic variables after the operation were compared with the baseline values using a paired t-test for within-group differences and repeated measures define. *P* values of 0.05 or less were considered statistically significant. All statistical analyses were performed using SPSS version 13 (SPSS Inc., Chicago, IL, USA) for Windows.

### 3.2. Ethics

The study protocol was approved with number 875 by ethics committee of Iran University of Medical Sciences at Iran University of Medical Sciences, Tehran, Iran.

## 4. Results

In this study, 60 patients who underwent orthopedic surgeries on lower limbs with spinal anesthesia were divided into two groups each receiving Ringer`s lactate or Hetastarch solutions during their surgeries. The mean age of studied patients was 34.1 ± 8.5 years with the range of 21-55 years. The mean age of patients in Ringer`s lactate and Hetastarch groups were 33.8 ± 8.5 years and 34.3 ± 8.5 years, respectively. The study groups did not have statistically significant difference for mean age, duration of surgery, and sex distribution (*P* > 0.05) (Table 1). Heart rate (HR), cardiac output (CO), stroke volume (SV), index of cardiac contractility (Icon), cardiac index (CI), and systemic vascular resistance (SVR) were measured before intravenous fluid preloading as baseline for defining further changes. The baseline values of mentioned variables did not show a significant difference between two groups using t-test (*P* > 0.05) (Table 2). Tables 3 and 4 show changes of HR, CO, SBP, diastolic blood pressure (DBP), SV, Icon, CI, and SVR in each 10 minutes from initiation of surgery to its end comparing with baseline values. Results showed that there were no significant differences between two groups about hypotension whereas cardiac index and index of contractility were significantly higher in Hetastarch group (Diagrams 1 to 8) (*P* < 0.05). Incidence of hypotension in two groups was not statistically significant (Table 5) (*P* > 0.05).

**Table 1.** Comparison of Mean Age and Sex Frequency in Patients Undergoing Orthopedic Surgery on Lower Limb with Spinal Anesthesia in Two Treatment Groups: Ringer`s Lactate and Hetastarch

	Ringer`s lactate (n = 30)	Hetastarch (n = 30)	*P* value
**Age, y, Mean ± SD**	33.8 ± 8.5	34.3 ± 8.5	0.81 ^a^
**Sex**			
**Male**	20 (66.7%)	24 (80%)	0.19 ^b^
**Female**	10 (33.3%)	6 (20%)	
**Duration of the surgery, min**	82.3 ± 12.2	76.0 ± 17.3	0.10 ^a^

^a^t-test

^b^Chi-Square

**Table 2.** Comparison of Means of HR, CO, SBP, DBP, SV, Icon, and SVR in Two Treatment Groups before Surgery (Baseline Values)

	Ringer`s lactate (n = 30)	Hetastarch (n = 30)	*P* value
**HR, /min**	81.9 ± 17.6	86.5 ± 14.9	0.28 ^a^
**CO, lit/min**	5.7 ± 1.6	6.5 ± 2.3	0.11 ^a^
**SBP, mmHg**	118.1 ± 14.7	115.7 ± 18.3	0.58 ^a^
**DBP, mmHg**	72.7 ± 14.0	71.7 ± 13.7	0.78 ^a^
**SV, lit**	70.1 ± 18.1	76.6 ± 23.6	0.23 ^a^
**CI, m^^2^/lit/min**	3.1 ± 0.8	3.5 ± 1.2	0.10 ^a^
**Icon, mmHg/s**	55.1 ± 25.2	60.1 ± 21.2	0.40 ^a^
**SVR, cm^^-5^/Dynes/sec**	0.39 ± 0.06	0.39 ± 0.09	0.85 ^a^

Abbreviations: CI, Cardiac Index; CO, Cardiac Output; DBP, Diastolic Blood Pressure; HR, Heart Rate; Icon, Index of Cardiac contractility; SBP, Systolic Blood Pressure; SV, Stroke Volume ; SVR, Systemic Vascular Resistance.

^a^t-test.

**Table 3.** Changes of HR, CO, SBP, and DBP in Comparison with the Baseline Values in Each 10 Minutes up to the end of Surgery

	CO (SD ± Mean)	*P* value	HR (SD ± Mean)	*P* value	SBP (SD ± Mean)	*P* value	DBP (SD ± Mean)	*P* value
**Before surgery**								
Ringer`s lactate (n = 30)	1.6 5.7	0.09	17.6 ± 81.9	< 0.001	14.7 ± 118.1	0.57	14.0 ± 72.7	0.78
Hetastarch (n = 30)	2.4 ± 6.6		14.9 ± 86.5		18.3 ± 115.7		13.7 ± 71.7	
**10 min after surgery**								
Ringer`s lactate (n = 30)	1.6 ± 5.2	0.42	16.6 ± 80.3	0.82	15.3 ± 116.8	0.80	11.3 ± 70.6	0.52
Hetastarch (n = 30)	2.1 ± 6.2 ^b^		13.8 ± 81.2 ^a^		18.5 ± 117.9		11.8 ± 72.5	
**20 min after surgery**								
Ringer`s lactate (n = 30)	1.6 ± 5.1 ^a^	0.02	17.6 ± 77.7	0.71	16.2 ± 116.5	0.96	12.9 ± 70.3	0.52
Hetastarch (n = 30)	2.1 ± 6.2 ^b^		13.2 ± 79.2 ^a^		20.8 ± 116.3		14.6 ± 72.6	
**30 min after surgery**								
Ringer`s lactate (n = 30)	1.4 ± 5.1 ^a^	0.06	13.8 ± 76.1 ^a^	0.67	17.7 ± 118.0	0.74	11.0 ± 71.7	0.69
Hetastarch (n = 30)	2.2 ± 6.0 ^a^		14.0 ± 77.6 ^a^		18.0 ± 116.5		12.5 ± 72.9	
**40 min after surgery**								
Ringer`s lactate (n = 30)	1.3 ± 4.8 ^a^	0.002	12.7 ± 74.0 ^a^	0.18	13.5 ± 115.9	0.29	10.5 ± 69.5	0.06
Hetastarch (n = 30)	2.3 ± 6.3		15.1 ± 78.8 ^a^		23.0 ± 121.1		13.8 ± 75.4	
**50 min after surgery**								
Ringer`s lactate (n = 30)	1.2 ± 4.9 ^a^	0.002	12.7 ± 74.6 ^a^	0.04	12.5 ± 115.3	0.24	1.6 ± 68.2	0.02
Hetastarch (n = 30)	1.9 ± 6.2 ^b^		15.0 ± 81.9		20.5 ± 120.4		14.2 ± 74.2	
**60 min after surgery**								
Ringer`s lactate (n = 30)	1.3 ± 5.0 ^a^	0.01	13.7 ± 87.7	0.01	15.7 ± 116.4	0.41	11.4 ± 69.2	0.10
Hetastarch (n = 30)	1.9 ± 6.1 ^b^		18.8 ± 77.5		18.9 ± 120.1		13.5 ± 74.5	
**70 min after surgery**								
Ringer`s lactate (n = 30)	1.4 ± 5.2	0.20	12.9 ± 79.9	0.40	16.2 ± 118.1	0.59	11.3 ± 70.8	0.18
Hetastarch (n = 30)	1.6 ± 5.7		15.6 ± 83.0		20.0 ± 120.6 ^a^		15.0 ± 75.4	
**80 min after surgery**								
Ringer`s lactate (n = 30)	1.3 ± 5.4	0.03	14.1 ± 79.6	0.09	12.6 ± 114.3	0.04	9.5 ± 69.3	0.002
Hetastarch (n = 30)	1.6 ± 6.2		19.7 ± 87.2		27.2 ± 125.5		13.5 ± 79.0 ^ab^	
**90 min after surgery**								
Ringer`s lactate (n = 30)	1.6 ± 5.5	0.21	13.2 ± 81.8	0.03	14.0 ± 114.2	0.002	12.1 ± 71.2 ^ab^	0.002
Hetastarch (n = 30)	1.5 ± 6.0		16.5 ± 90.0		22.6 ± 129.5 ^a^		12.1 ± 83.5 ^ab^	

Abbreviations: CO, Cardiac Output; DBP, Diastolic Blood Pressure; HR, Heart Rate; SBP, Systolic Blood Pressure.

^a^*P* value < 0.05- compared to the baseline.

^b^*P* value < 0.05, between two groups.

**Table 4.** Changes of SV, Icon, and SVR in Comparison with the Baseline Values in Each 10 Minutes up to the End of Surgery

	SV (Mean ± SD)	*P* value	Icon (Mean ± SD)	*P* value	CI (Mean ± SD)	*P* value	SVR (Mean ± SD)	*P* value
**Before surgery**								
Ringer`s lactate (n = 30)	1.18 ± 1.7	0.002	2.25 ± 1.55	0.92	8 ± 1.3	< 0.001	6 ± 39	0.76
Hetastarch (n = 30)	6.23 ± 6.76		2.21 ± 1.6		2.1 ± 6.3		9 ± 39	
**10 min after surgery**								
Ringer`s lactate (n = 30)	1.18 ± 2.67	0.002	0.2 ± 5 ^ab^	0.04	7 ± 9.2 ^ab^	0.002	8 ± 37	0.68
Hetastarch (n = 30)	6.20 ± 8.75		0.22 ± 2.59		1.1 ± 4.3		9 ± 38	
**20 min after surgery**								
Ringer`s lactate (n = 30)	4.18 ± 2.67	0.09	3.20 ± 8.48 ^a^	0.013	7 ± 8.2 ^ab^	0.0009	6 ± 38	1.00
Hetastarch (n = 30)	2.21 ± 5.76		9.17 ± 9.57		1.1 ± 4.3		6 ± 39	
**30 min after surgery**								
Ringer`s lactate (n = 30)	3.18 ± 7.69	0.98	8.22 ± 6.51	0.001	8 ± 9.2 ^ab^	0.003	6 ± 37	0.91
Hetastarch (n = 30)	3.22 ± 5.76		2.22 ± 7.58		2.1 ± 5.3		7 ± 38	
**40 min after surgery**								
Ringer`s lactate (n = 30)	7.16 ± 9.66 ^b^	0.019	8.20 ± 7.48 ^a^	0.034	8 ± 8.2 ^ab^	0.002	7 ± 37	0.91
Hetastarch (n = 30)	2.21 ± 5.78		4.26 ± 6.59		2.1 ± 6.3		6 ± 37	
**50 min after surgery**								
Ringer`s lactate (n = 30)	6.19 ± 2.65 ^a^	0.002	4.22 ± 8.49 ^a^	0.003	7 ± 7.2a ^b^	0.196	6 ± 37	1.00
Hetastarch (n = 30)	0.18 ± 7.74		9.18 ± 2.55		9 ± 4.3		6 ± 37	
**60 min after surgery**								
Ringer`s lactate (n = 30)	7.15 ± 0.66 ^a^	0.96	0.21 ± 0.47	< 0.001	7 ± 7.2 ^ab^	0.0006	6 ± 37	1.00
Hetastarch (n = 30)	7.17 ± 2.74		9.19 ± 9.52		1.1 ± 5.3		6 ± 36	
**70 min after surgery**								
Ringer`s lactate (n = 30)	8.20 ± 3.67	0.15	1.22 ± 4.52 ^a^	< 0.001	7 ± 0.3	0.20	7 ± 38	1.00
Hetastarch (n = 30)	6.17 ± 6.7		6.22 ± 1.52		8 ± 4.3		7 ± 36	
**80 min after surgery**								
Ringer`s lactate (n = 30)	5.18 ± 2.71	0.001	8.20 ± 7.35 ^a^	0.0006	6 ± 0.3	0.0003	7 ± 38	0.76
Hetastarch (n = 30)	3.14 ± 1.7		3.17 ± 0.5		9 ± 4.3		10 ± 39	
**90 min after surgery**								
Ringer`s lactate (n = 30)	7.17 ± 5.70	< 0.001	5.21 ± 6.54	0.11	7 ± 0.3	0.103	7 ± 38	0.76
Hetastarch (n = 30)	2.17 ± 0.69		3.17 ± 2.49		8.0 ± 3.3		10 ± 40	

Abbreviations: Icon, Index of Cardiac contractility; SV, Stroke Volume; SVR, Systemic Vascular Resistance.

^a^*P* value < 0.05- compared to the baseline.

^b^*P* value < 0.05, between two groups.

**Table 5.** Comparison of Incidence of Hypotension in Patients Undergoing Orthopedic Surgery on Lower Limb With Spinal Anesthesia in two Treatment Group: Ringer`s Lactate and Hetastarch

	Ringer`s Lactate, (n = 30)	Hetastarch, (n = 30)	*P* value
**Hypotension**			
Yes, No. (%)	50 (16.7%)	4 (13.3%)	0.50 ^a^
No, No. (%)	25 (83.3%)	26 (86.7%)	Non-Significant

^a^Chi-Square.

## 5. Discussion

In this study, patients were randomly divided into two groups: Ringer`s lactate or Hetastarch. Based on our results, two groups did not have statistically significant difference for mean age, surgery duration, and sex distribution. Our findings showed that there were no significant differences between two groups about hypotension, whereas cardiac index and index of contractility were significantly higher in Hetastarch group. Sympathetic block during spinal anesthesia led to changes in cardiac indexes like blood pressure, CO, and HR that finally led to relative hypovolemia and decline in venous return. Results of some studies have shown that prophylactic administration of crystalloids had no effect on prevention from spinal anesthesia-induced hypotension in patient undergoing surgeries (4, 5). Several studies about electro-velocimetry set monitoring revealed reliability of its outcome compared to invasive methods for cardiac status assessment like transpulmonary thermodilution (8) and transesophageal electrocardiography (12). Moreover, this assessment method had a good correlation with golden standard method for cardiac index (direct oxygen FIK method) (11, 14). Since about 75% of all types of crystalloids diffuse in intracellular spaces, their efficiency in increasing plasma volume is transient (11). Although administration of crystalloid is appropriate in most of patients, their administration may be inefficient in some specific groups like patients with renal problems or congestive heart failure. Also, sometimes, excess administration of crystalloids may lead to peripheral and pulmonary edemas, and has a low effect on plasma volume. Preloading with crystalloid or colloid solutions has been widely recommended for prevention from hypotension during spinal anesthesia (15). Since colloid solutions remain longer in the vessels due to their physical specificity, theoretically they are logical choice to prevent from hypotension during spinal anesthesia. Hetastarch 6% is a synthetic colloid solution with average molecular weight of 450,000, pH = 5.5, and osmolarity of 310 mOsm/Lit with its oncotic pressure similar to serum (34 mm Hg). Its half-life in the vessels is 25.5 hour and has the capacity to increase plasma volume to more than administration value (16). The result of present study on patients undergoing elective femoral fractures surgeries who received Hetastarch or Ringer`s lactate solutions showed that both solutions had the same effects on compensation of hypotension, CI, and CO in patients undergoing spinal anesthesia. After spinal anesthesia, CO can be held using maneuvers like Trendelenburg position or infusion of Ringer`s lactate or Hetastarch solutions (17). Results of Zorko N *et al*. about effects of Hetastarch and Ringer`s lactate solutions and changing patient`s position to Trendelenburg position on CO in 70 patients with ASA I-II under orthopedic surgeries showed that administration of Ringer`s lactate and Hetastarch solutions was effective to prevent from decline in CO during sympathetic block subsequent to spinal anesthesia, but Hetastarch solution could hold this situation with more stability during the surgery (17). Sharma SK *et al*. also confirmed that 500 mL of Hetastarch solution was more effective than 2000 mL of Ringer`s lactate solution in preventing from spinal anesthesia-induced hypotension. Meanwhile, similar results have been revealed in studies by Madi-Jebara S and Ueyama H *et al*. which implied much effectiveness of Hetastarch solution in prevention from changes in cardiac indexes in patients undergoing spinal anesthesia (18, 19). In other study by Marhofer P *et al*., it was illustrated that spinal anesthesia-induced hypotension in old patients with ASA III did not change due to decrease in SVR and cardiac indexes (20). In this study, however, incidence rate of hypotension was lower in Hetastarch group, but this difference was not significant (16.7% in Ringer`s lactate group vs. 13.3% in Hetastarch group, *P* = 0.5). In Buggy D *et al*. study, these values were 39% and 62% in groups receiving crystalloid and colloids, respectively, which was in contrast with our results (21). Nephrotoxicity has been reported as the probable side effect of synthetic colloids. As evaluation of probable side effects of colloids was not within the scope of our study, this issue was not assessed; this may be considered as one of our study limitations. Also, such adverse effect was not reported in similar previous studies (15-17, 19). The rate of occurrence of hypotension was not different. In this regard, there was significant difference between two groups in the setting of cardiac index and contractility index from 10 minutes after beginning of operation. In our study, we found that there was no difference between Ringer's lactate and Hetastarch solutions in the setting of blood pressure controlling, but the stability of other cardiac indexes including contractility index, cardiac index, and cardiac output were significantly better controlled by Hetastarch solution compared to Ringer's lactate solution. Therefore, it could be brought up that there was no preference in the setting of preloading between these two solutions. In spite of that, more studies are needed to prove the preference of Hetastarch to Ringer's lactate solutions.
